# Are Humans Too Generous and Too Punitive? Using Psychological Principles to Further Debates about Human Social Evolution

**DOI:** 10.3389/fpsyg.2016.00799

**Published:** 2016-05-27

**Authors:** Max M. Krasnow, Andrew W. Delton

**Affiliations:** ^1^Evolutionary Psychology Laboratory, Department of Psychology, Harvard University, CambridgeMA, USA; ^2^Department of Political Science and College of Business, State University of New York at Stony Brook, Stony BrookNY, USA

**Keywords:** evolutionary psychology, ecological rationality, cooperation, trust, punishment

## Abstract

Are humans too generous and too punitive? Many researchers have concluded that classic theories of social evolution (e.g., direct reciprocity, reputation) are not sufficient to explain human cooperation; instead, group selection theories are needed. We think such a move is premature. The leap to these models has been made by moving directly from thinking about selection pressures to predicting patterns of behavior and ignoring the intervening layer of evolved psychology that must mediate this connection. In real world environments, information processing is a non-trivial problem and details of the ecology can dramatically constrain potential solutions, often enabling particular heuristics to be efficient and effective. We argue that making the intervening layer of psychology explicit resolves decades-old mysteries in the evolution of cooperation and punishment.

## What Theories Do We Need to Explain Human Cooperation?

For decades, psychologists and other behavioral researchers have used a suite of well-understood theories from evolutionary biology to map human psychology. To understand the logic of families, parents, and siblings, evolutionary biologists developed kin selection theory (aka inclusive fitness theory), a formal, mathematical theory describing under what circumstances an animal should pay a personal cost to provide benefits to another animal, depending on how closely they are genetically related (Hamilton, 1964). Psychologists have borrowed this theory and applied it to humans, testing whether and how human psychology is sensitive to cues of relatedness (DeBruine, 2002; Lieberman et al., 2007). To understand trade, exchange, and other acts of cooperation, evolutionary biologists developed the theory of direct reciprocity, a formal theory describing how animals should trade benefits for mutual gain over time based on, among other things, how long the relationship will last (Trivers, 1971). Borrowing this theory, psychologists have tested under what conditions humans will reciprocate (Cosmides and Tooby, 2005). Evolutionary biology has also developed theories about reputation and gossip: indirect reciprocity and biological markets are formal theories describing how animals should behave given that reputations exist (Noë and Hammerstein, 1995; Nowak and Sigmund, 2005; Barclay, 2013).

Nonetheless, there has been strenuous disagreement about how to interpret behavioral data on human cooperation. In particular, researchers have questioned whether humans are more generous and more punitive than predicted by the classic theories outlined above. If so, then different theories, like genetic or cultural group selection, are needed. We do not think that such a move is necessary; instead, we think that the classic theories suffice to explain known behavior. In brief, our argument is that, in the domain of cooperation, researchers have too often failed to appreciate the well-known distinction between the ultimate level of selection pressures and the proximate, surface level of evolved psychology (for analyses of this distinction, see Symons, 1992; and Tooby and Cosmides, 1992). Because of this, the literature on human social evolution has been dogged by mysterious gulfs between theoretical prediction and empirical evidence where none should exist.

Why should thinking in terms of proximate psychology be able to help in this way? Because theories of social evolution *are* theories of psychology—what else is social evolution but the evolution of psychological mechanisms for sociality? And if interpretations of data do not accurately take into account how real minds, operating in real time, with imperfect information, actually function, then claims that classic theories are not sufficient may be unwarranted. So, are humans too generous and too punitive?

## Are Humans Too Generous?

For decades, evolutionary thinkers have been puzzled by humans’ “irrational” generosity. We often are nice to perfect strangers—people who are not kin and who we will never see again. In the real world this would include tipping at a restaurant when traveling abroad to a place you never plan to visit again. Why tip? It is not legally required. You and the waiter are not close kin. You will never see him again, so reciprocity is impossible. And because no one you know is around, your reputation is safe. No standard theory of social evolution seems to apply, but many of us tip nonetheless.

Similar results occur in the lab. Consider the economic dictator game: two strangers interact anonymously and just once. One person, the dictator, unilaterally divides a stake of real money between herself and the other person. They are not close kin. They interact only once, so there is no potential for reciprocity. And the interaction is anonymous, so there is no potential for cultivating a reputation. Yet just as you were willing to tip, dictators are often generous (Camerer, 2003).

Because standard theories of social evolution appear ruled out, but cooperative behavior remains, theorists from evolutionary anthropology, biology, and economics developed new theories of human evolution based on genetic or cultural group selection frameworks (Gintis, 2000; Fehr and Henrich, 2003; Henrich, 2004). These theories require that much of human cooperation is a product of culturally specific norms and imply that generosity and cooperation are not universal features of human nature (Richerson et al., 2016). These evolutionary theories have recently found their way into prominent theories of psychology (Haidt, 2012). However, whereas theories of kin selection, reciprocity, and reputation are well-understood and have countless empirical successes (Buss, 2005), theories of group selection are theoretically controversial (West et al., 2007) and it is not clear they have ever made unique, empirically confirmed predictions (Krasnow and Delton, 2016).

We think the mystery can instead be parsimoniously solved by distinguishing the surface logic of the proximate psychology from the deep logic of the ultimate selection pressures that created this psychology. While biological theory provides tools for understanding the latter, psychology is best positioned to make sense of the former. For instance, the psychology of sexual attraction evolved to find reproductively viable partners; that is its deep logic. But the surface logic of the proximate psychology can only use information that was reliably available in the human ancestral past, such as age, health, and other physical appearance cues. This explains what might otherwise be puzzling: why are men attracted to women on birth-control—women who are not presently reproductively viable? The answer is that contraception is a modern invention; men’s proximate psychology was not designed for a world where such technology was available. Similarly, the deep logic of many of our food preferences is to find calorie-dense food. Because these were scarce ancestrally, our proximate psychology causes us to eat far more than necessary, contributing to the modern obesity epidemic. Our work applies this approach to the study of social behavior, asking: “What should a proximate psychology for, e.g., direct reciprocity look like?”

Our analysis starts with two assumptions. First, we assume that, through individual selection for direct reciprocity, humans have an evolved psychology for reciprocity and exchange (Cosmides and Tooby, 2005; see Nowak, 2006 on how reciprocity can robustly evolve); this is an assumption made even by proponents of group selection (see Fehr and Henrich, 2003 p. 61; Gintis et al., 2003 p. 155). Second, we assume uncertainty: real-world decisions are always made with imperfect, noisy information. The mind’s decision-making processes must necessarily reflect this uncertainty. In cooperation, this uncertainty is rampant: is your behavior private or observed? Would you gain from the trade or not? Is the person you meet now someone you will see again? Even if cooperation would pay for both parties, formal models predict that if you know for certain the relationship is one-shot (that is, you interact once and only once) then you should never cooperate; if the relationship is certainly repeated, however, you should cooperate (Trivers, 1971; Axelrod and Hamilton, 1981). But of course you can almost never know this with certainty.

More critically, uncertainty entails errors, the costs of which may not be equal (Haselton and Buss, 2000; Yamagishi et al., 2007; for a discussion of this in biology, see Johnson et al., 2013). There are two possible errors here: cooperating when you will actually interact with your partner only briefly, and defecting when you will actually see your partner many times. The costs are not equal. If you erroneously cooperate, you have given up a little for no gain. But if you erroneously defect, you might miss out on a life-long, mutually beneficial relationship. Given this asymmetry, evolution should create human minds biased to cooperate, even if cooperation does not seem rationally warranted—you seldom know for sure you won’t see someone again, so play it safe and cooperate.

And that is exactly what we found: in agent-based simulations of a human-like ecology, natural selection favored agents willing to cooperate even with partners they would never see again (Delton et al., 2011b). This was true over the vast majority of parameter combinations we tested, involving many variations of the costs and benefits of cooperation, lengths of interactions if they are repeated, and the *a priori* probabilities of interactions being repeated or one-shot. Notably, the (very small) parameter space where cooperation in one-shot encounters did not evolve was largely the same as the parameter space that did not favor cooperation even in repeated interactions. When cooperation pays in repeated interactions, it pays in situations that are likely to be one-shot as well.

Our simulation therefore provides a straightforward explanation for “irrational” generosity, without invoking group selection—as no group selection was possible in this simulation yet one-shot generosity evolved nonetheless. Moreover, our simulation ignored the possibility that others could learn of agents’ behavior; had it been possible for reputations to spread beyond the dyad, the results would only have been stronger. By distinguishing the proximate psychology from the ultimate selection pressures, this research illustrates one way the ruthless process of natural selection can craft psychologies that are generous, cooperative and trusting (see also Krasnow et al., 2013; for allied approaches see Barclay, 2011; Krupp and Taylor, 2015).

Whenever the best response is determined by a noisy cue and the costs of decision errors are asymmetrical, the proximate psychology should be designed to avoid making the costly error (here, defecting in a repeated relationship) at the expense of making lots of cheap errors (lots of “irrational” generosity). Although evolutionary theorists have long used decision-making tools to understand behavior (e.g., Giraldeau, 1997; Sherman et al., 1997), this approach had not been extended to the long standing problem of one-shot cooperation. By using a psychology-inspired approach to decision making, we were able to develop a new way of looking at an old debate and solve an enduring mystery (for replies see McNally and Tanner, 2011; Zefferman, 2014; for rejoinders see Delton et al., 2011a; Delton and Krasnow, 2014).

## Are Humans Too Punitive?

Punishment is the stick to cooperation’s carrot. Many animals use punishment to try to change others’ behavior to achieve beneficial outcomes (Clutton-Brock and Parker, 1995) and such theories of punishment-as-bargaining have a long history in the biological (Hammerstein and Parker, 1982) and social sciences (Schelling, 1980). This is punishment’s deep logic. But, just as with generosity, when human punishment has been studied, many researchers have concluded that humans are “irrationally” punitive—often punishing with no apparent incentive. As with generosity, these conclusions come largely from studies of one-shot, anonymous punishment (Fehr et al., 2002). And again, researchers turned to theories of group selection for answers (Henrich et al., 2010). But does the puzzle of irrational punishment even exist? We think the answer again is no. Punishment in conditions with no rational incentive only violates the deep logic of punishment. To answer this question about the surface logic we must ask, “What should a proximate psychology for punitive bargaining look like?”

The surface logic of our evolved psychology can only reflect the long-term regularities of our ancestral past. A general implication of this is that care must be taken when interpreting experiments of anonymous punishment (Hagen and Hammerstein, 2006). Showing that men are still attracted to women on birth-control—an evolutionarily anomalous technology—does not imply that this mechanism isn’t an adaptation for preferring reproductive viability; showing that punishment still occurs in one-shot anonymous encounters—an evolutionarily anomalous situation—similarly does not imply that this mechanism isn’t an adaptation for bargaining for better treatment.

Instead of simply testing for anonymous punishment, studies must test the fit between features of our evolved punishment psychology and ancestral ecological regularities. One such regularity is that punishment is costly: punishment takes time and energy, and there is always the possibility of retaliation. So, punishment should be contingent on ancestral cues of cost effectiveness: is it easy for me to punish because, for example, I am physically formidable or socially connected? When the answer is yes, people are more likely to aggress to get their way (Von Rueden et al., 2008; Sell et al., 2009). Is this person likely to offend against me or people I value if I fail to punish them? When people believe the answer is yes, they are more likely to punish (Krasnow et al., 2016). Will punishing a particular person give me a reputation as someone willing to defend his own interests? If so, people are more likely to punish (Benard, 2013). Is the person treating me poorly someone who might be valuable for cooperation in the future? When people intend to cooperate with someone in the future, they are more likely to punish their bad behavior in the present (Krasnow et al., 2012).

Punishment has been especially mysterious in groups: even if punishment is beneficial, why should I bear its costs instead of leaving them to others in the group (Yamagishi, 1988; Boyd and Richerson, 1992)? Previous work identified this “second-order free riding” as a fatal barrier to the evolution of punishment in groups: punishment benefits the entire group, so why should any particular person pay the cost of providing it? Because people regularly do punish even in sizable groups, many researchers have concluded group selection is needed. We think this is premature. We have modeled how a basic psychological insight solves the problem (Krasnow et al., 2015). People vary quantitatively in pretty much any psychological trait you can think of. This should include their punitive tendencies. Yet past models of group cooperation and punishment restricted people to distinct types: either you are a punisher or you are not.

Once we allowed evolution to work on quantitative individual differences, the problem was solved: evolution easily and reliably created organisms with some willingness to punish (including in groups of up to 25, the largest group size we tested). When everyone in a group is a little willing to punish, this means that at any given moment, someone will punish bad behavior. These results obtained under reasonable ecological assumptions: that punishment recalibrates non-cooperators to cooperate (preventing re-offense against the punisher), that life is long enough that this later cooperation repeats (raising benefits), and that punishment is probabilistic rather than all-or-none (lowering costs). Importantly, although the simulation allowed for the possibility of second-order free riding, agents nonetheless evolved to be punitive and this punishment sustained cooperation; second-order free riding was not an impediment to the evolution of group cooperation here. And all this occurred in a simulation where group selection was not possible (Krasnow et al., 2015).

As with generosity, examining the surface logic of our evolved psychology has helped address a long standing debate about the evolution of punishment in groups. Basic psychology helped to solve another decades-long mystery in the biological sciences.

## Psychology As the Missing Piece

Why did researchers argue for decades that if humans had adaptations for direct reciprocity they should defect in one-shot, anonymous experiments? Why did the second-order free-rider problem appear to be so insurmountable? We argue that this is the reliable result of failing to properly consider the role of psychology as the intervening level of analysis between the evolutionary game theory of the selection pressures and the behavior that ultimately results from them. By inserting even a minimally plausible psychology, both mysteries evaporate. While we focus on research on cooperation and punishment here, this argument should apply generally. Theories of animal behavior are theories of psychology. Theoretical biology has had tremendous success leveraging analyses of the deep logic of selection pressures into predictions of organismal design. By contrast, especially in debates on the evolution of human cooperation, less attention has been paid to the fact that this deep logic must be played out by a surface logic tuned to the information structure of a species’ ancestral ecology. This is the role of psychology in evolutionary science. Here we show how off course the science can get when this old point is not remembered.

## Conflict of Interest Statement

The authors declare that the research was conducted in the absence of any commercial or financial relationships that could be construed as a potential conflict of interest.

## Author Contributions

MMK and AWD both conceived of and wrote this article together.
